# A practical microreactor for electrochemistry in flow

**DOI:** 10.3762/bjoc.7.127

**Published:** 2011-08-15

**Authors:** Kevin Watts, William Gattrell, Thomas Wirth

**Affiliations:** 1 Cardiff University, School of Chemistry, Park Place, Cardiff CF10 3AT, UK; 2 Prosidion Ltd, Watlington Road, Oxford OX4 6LT, UK

**Keywords:** diaryliodonium compounds, electrochemistry, flow chemistry, microreactor

## Abstract

A microreactor for electrochemical synthesis has been designed and fabricated. It has been shown that different reactions can be carried out successfully using simple protocols.

## Introduction

Electrochemical reactions offer a clean route to the formation of anion and cation radical species from neutral organic molecules. Electrons can be added or removed from the substrates under mild conditions without the need for chemical oxidizing or reducing reagents which might complicate the reaction sequence [1]. A major advantage of electrochemical methods is the reduced formation of side products, as no chemical reagents are necessary. Electrochemical methods therefore provide a better environment for subsequent reactions involving the electrochemically generated reactive species [2]. Traditional electrochemical batch methods can suffer from several drawbacks. The electrical field in the cell is heterogeneous and often supporting electrolytes have to be used, which must be removed after the reaction. As a result, only a few electrochemical processes for the production of organic compounds have been commercialized [3]. In microreactors, the distances between electrodes can be very small such that the two diffusion layers of the electrodes overlap or become "coupled". This allows ions to be electrogenerated and play the role of the supporting electrolyte.

Different microreactor systems have already been developed for chemistry in this area and have been successfully employed in the conduction of electrochemical reactions without any added electrolyte [4–5]. Atobe et al. constructed a thin layer flow cell from platinum and/or glassy carbon plates (3 × 3 cm), separated by adhesive tape (80 µm), with a space left in between to act as the channel, and the devices were sealed with epoxy resin [6]. They reported the self-supported, paired electrosynthesis of 2,5-dimethoxy-2,5-dihydrofuran from furan with excellent yields and flow rates of up to 0.5 mL·min^−1^. A microflow system where the current flow and liquid flow are parallel was reported by Yoshida et al. [7]. Two carbon fibre electrodes were separated by a hydrophobic porous PTFE membrane (75 µm thickness). The substrate solution was fed into the anodic chamber and flowed through the membrane into the cathodic chamber, where it would leave as products. The carbon fibre electrodes used in this design allow for a much greater surface area than the empty space between electrodes in the setup reported by Atobe. The anodic methoxylation of 4-methoxytoluene was carried out in the electrochemical cell under a constant current of 11 mA with a flow rate of 2 mL·h^−1^ resulting in a 90% conversion. A simpler configuration of electrodes was reported by Haswell et al. [8]. Two platinum electrodes with a surface area of 45 mm^2^ were positioned with an inter-electrode gap of either 160 µm or 320 µm, and C–C bond forming reactions based on the electro-reductive coupling of activated olefins and benzyl bromide derivatives were reported. The best result obtained was a 98% formation of the product at a flow rate of 10–15 µL·min^−1^ with a current of 0.6 mA.

**Scheme 1:**
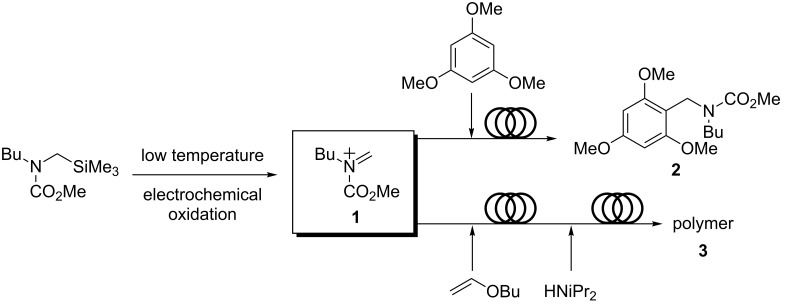
Electrochemically generated *N*-acyliminium ions **1** and subsequent reactions.

Yoshida reported a method of producing carbocations such as **1** in the absence of a suitable nucleophile (the "cation pool" method). This is an unconventional method because the ions generated are unstable and usually need to be trapped immediately after generation. In the "cation flow" method, a carbocation is generated continuously in a flow system by low temperature electrolysis. The generation of the cation can be monitored by a FTIR spectrometer in the flow system. The electrochemically generated *N*-acyliminium ions can then be used in different subsequent reactions (Scheme 1).

The first reaction studied was a Friedel–Crafts reaction of aromatic compounds. In comparison with the "cation pool" method, the "cation flow" method was far more successful for this reaction, producing the monoalkylated product **2** in 92% yield in contrast to the batch method, which led to a 1:1 mixture of mono- and dialkylation products [9]. The reaction in flow is complete within seconds, and the extremely fast 1:1 mixing in a micromixer, together with efficient heat transfer in the microsystem, seems to be responsible for the dramatic increase in the product selectivity. Cycloadditions using the *N*-acyliminium ions as heterodienes with a variety of dienophiles, such as alkenes and alkynes, give the corresponding [4 + 2] cycloaddition products in high yields [10]. The same authors also reported a successful polymer synthesis using the same technique by adding a monomer to the initiator (*N*-acyliminium ions) followed by micromixing and the addition of the terminator (diisopropylamine) in a subsequent micromixer. This was considered to be a superior technique to the batch method as the molecular weight distribution of the polymer **3** decreased in the flow system [11].

Yoshida et al. also reported the electrochemical iodination of aromatic compounds by elemental iodine followed by a subsequent reaction with aromatic compounds. This sequential method has one main advantage over the batch method, in that the polyiodination problem for highly reactive aromatic compounds, based on disguised chemical selectivity, can be avoided by the micromixing of a solution of preformed iodine cations (I^+^) and a solution of an aromatic compound, increasing the yield of mono iodination product from 38% to 85% [12]. Nishiyama et al. reported the synthesis of hypervalent iodine compounds using electrochemistry [13]. Electrochemical microreactors for investigations of laminar flow have also been reported recently [14–15].

## Results and Discussion

**Figure 1:**
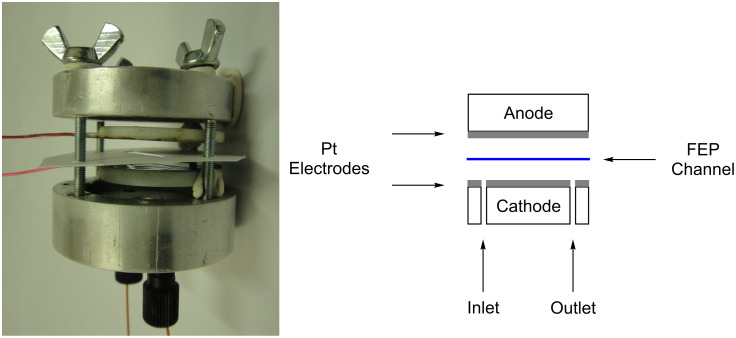
Electrochemical microreactor.

The target of this research is to develop a simple and practical microreactor in which to carry out electrochemical reactions in flow. A small gap between the electrodes, to avoid the necessity of electrolytes, and a simple and robust setup, were the leading principles in the design of the reactor. Herein we describe the construction of such a simple microreactor, some initial test reactions and the novel application to the continuous synthesis of diaryliodonium compounds. A microflow electrochemical reactor made out of two aluminium bodies (50 mm diameter, 25 mm height) was manufactured. The electrodes are constructed of two PTFE plates (35 mm diameter, 4 mm height) onto which 0.1 mm platinum foil electrodes [16] are mounted. The wires for connection to the potentiostat are fixed into the PFTE plate under the platinum. The electrodes are held apart by a FEP (fluorinated ethylene propylene) foil of variable thickness, into which a rectangular reaction channel is cut (3 × 30 mm) giving an overall channel volume of 23 µL (FEP foil 254 µm thick), and the whole device is held together by steel screws and wing nuts. The opened device is shown in Figure 1.

**Scheme 2:**
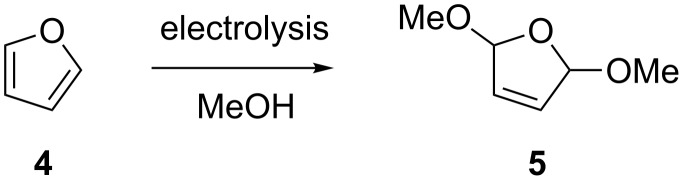
Electrolysis of furan.

Known electrochemical reactions were used as test reactions for the electrochemical microreactor. The oxidative methoxylation of furan **4** in methanol (Scheme 2) is a very clean reaction with no extra reagents necessary. The product **5**, obtained in an approximate 1:1 *syn*:*anti* ratio, was identified by ^1^H NMR and GC/MS and showed that our system functioned satisfactorily for electrochemical reactions. A yield for this reaction was not determined, but full conversion was achieved as established by GC/MS. The reaction shown in Scheme 2 has previously been performed in batch electrolysis leading to product **5** in 78% yield [17].

**Scheme 3:**
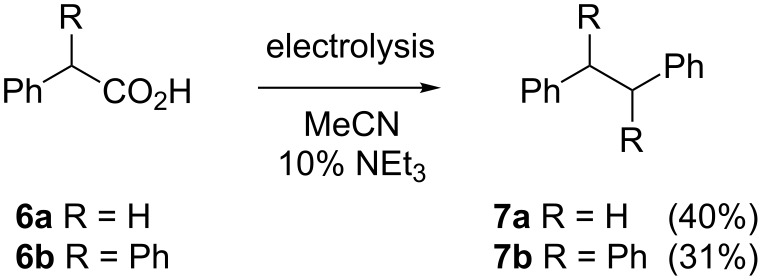
Kolbe electrolysis of phenylacetic acids **6** in flow.

In addition, Kolbe-type reactions were investigated in flow. 2-Phenylacetic acid (**6a**) was used as a substrate and yields of up to 40% of 1,2-diphenylethane (**7a**) were obtained with the device depicted in Figure 1; the reactions are shown in Scheme 3. The Kolbe reaction did not seem to be a very suitable reaction for a flow reactor due to the large amount of carbon dioxide and hydrogen that is formed at the anode during the decarboxylation reaction. This was partially overcome by neutralising the solution with triethylamine as base, hence carbon dioxide gas was not liberated during the reaction [18]. The reactions were carried out in acetonitrile instead of methanol to reduce the amount of side-products that could be formed due to the reduction of methanol at the cathode. The Kolbe electrolysis of **6a** has also been described as a batch reaction, with a solid base, providing the product **7a** in 44% yield [19]. This means that the reaction conditions in the electrochemical microreactor were comparable to batch synthesis. The reaction conditions described for **6a** were also successful for 2,2-diphenylacetic acid (**6b**) and even an asymmetric reaction product could be formed through a mixture of phenylacetic acid (**6a**) and diphenylacetic acid (**6b**), although in smaller yield.

Hypervalent iodine compounds can be used in organic syntheses as mild, non-toxic and highly selective reagents. Iodine(III) reagents with two carbon ligands are known as iodonium salts. These salts are attractive alternatives to oxidants and catalysts based on heavy metals, as they have similar properties to those of heavy metal complexes and can, therefore, be used in similar reaction pathways, and are beneficial for organic synthesis due to their low toxicity and cost [20].

Iodonium salts are currently being used in three main types of reactions, namely ligand exchange, reductive elimination and ligand coupling. This is due to their highly electron deficient nature and high dissociation rates that make them excellent leaving groups. Symmetric salts are usually more desirable than asymmetric compounds purely for the reason that this avoids problems that can occur with selectivity in aryl-transfers. If a diaryliodonium salt is asymmetric it is usually the more electron deficient ligand that is transferred [21].

The formation of these compounds usually follows a two-step procedure. The first step is the oxidation of an iodoarene, which can then take part in an acid-catalyzed coupling reaction with another aryl compound. However, there are now more one-pot procedures to diaryliodonium salts known that involve both oxidation and ligand exchange directly from the aryl and iodoarene starting materials. The electrochemical oxidation of an iodoarene in the presence of another arene provides a quite general and simple one-step approach to the synthesis of diaryliodonium salts [22].

**Scheme 4:**
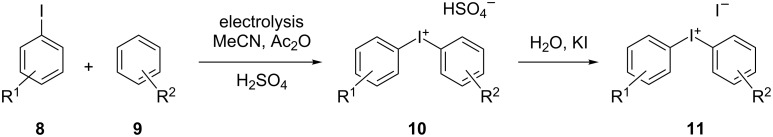
Synthesis of diaryliodonium salts **11** in flow.

We describe herein a simple procedure for the flow synthesis of diaryliodonium salts using the electrochemical microreactor device described above. The products were obtained in good yields and only minimal work-up was required after the reaction. The reaction takes place with an iodoarene that is oxidized at the anode. The radical cation then undergoes a reaction with the other arene to form an intermediate, followed by the loss of a second electron [23]. The solvent system consisted of acetonitrile, sulfuric acid (2 M) and acetic anhydride, which was reported to increase the selectivity of the coupling reaction [21]. The sulfuric acid acts both as a counter reaction to the oxidation at the anode, with proton reduction, and simultaneously provides a counter ion for the positively charged iodonium salt. It also acts as an electrolyte in the reaction described above. A general procedure for the synthesis of both, symmetric and asymmetric iodonium salts, was developed, as shown in Scheme 4, and yields of up to 72% were obtained.

Initially, the conditions of the reaction were optimized with iodotoluene **8a** (R^1^ = 4-Me) and toluene (**9a**) (R^2^ = Me). A current of 30 mA led to a yield of 72%, whereas with higher currents (35, 40, or 45 mA) the yield dropped to 50% for identical flow rates. The increased formation of (diacetoxyiodo) arene derivatives as side products was observed in these cases. In Table 1 the results of the experiments using different combinations of iodoarenes **8** and arenes **9** are summarized.

**Table 1:** Products and yields in the electrochemical generation of diaryliodonium compounds performed in an electrochemical microreactor (30 mA) at a flow rate of 80 µL/min (residence time: 17 s) at 25 °C.

Entry	R^1^	R^2^	Product	Yield [%]

1	4-Me	Me	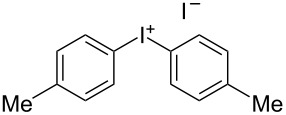 **11a**	72
2	4-Me	Et	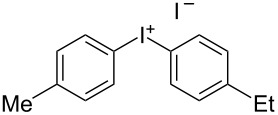 **11b**	51
3	4-Me	iPr	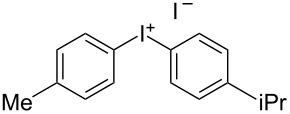 **11c**	60
4	H	Me	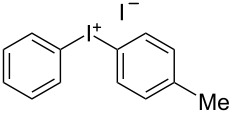 **11d**	44
5	H	Et	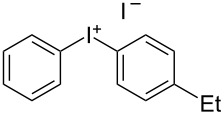 **11e**	39
6	H	iPr	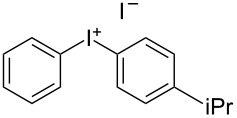 **11f**	19
7	H	*t*-Bu	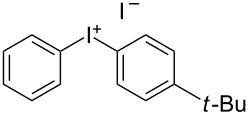 **11g**	64
8	3-Me	Me	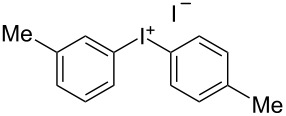 **11h**	36
9	3-Me	Et	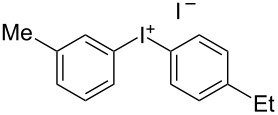 **11i**	25
10	3-Me	iPr	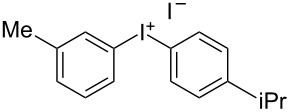 **11j**	18

Various yields were obtained in these experiments and not all reaction conditions were optimized. In some reactions the conversion was not complete and longer reaction times would have been necessary to achieve higher yields. Depending on the nature of the iodoarene **8**, traces of the corresponding diacetoxyiodo compounds were also observed as side products. After the reaction, treatment of the reaction mixture with potassium iodide was required, as the resulting diaryliodonium iodides **11** are insoluble in the reaction mixture (in contrast to the diaryliodonium hydrogensulfates **10**) and can thus be easily separated and purified by filtration and washing.

## Conclusion

The successful development of a microreactor for different types of electrochemical reactions is described. The device has the advantage of being very easily dismantled, allowing cleaning when reactions cause a blockage in the channel. It is also possible to change the internal volume of the channel quickly by changing the channel height through the foil thicknesses.

## Experimental

**General:** Melting points were obtained in open capillary tubes and are uncorrected. ^1^H NMR and ^13^C NMR spectra were recorded on a AV-400 Bruker spectrometer, in the solvents indicated, at 400 and 100 MHz, respectively. Mass spectra (*m*/*z*) and HRMS were recorded under the conditions of electron impact (EI). All reactions were monitored by thin-layer chromatography which was performed on precoated sheets of silica gel 60. The galvanostatic reactions were performed with a HEKA 510 galvanostat/potentiostat. The electrodes were cleaned with acetone after each reaction; no polishing was required. However, with earlier reactions using KF as an electrolyte in solution the electrodes did need polishing as they were slightly blackened. Methanol and acetonitrile were dried over 4 Å molecular sieves. All other chemicals were used as purchased without further purification.

### 

#### Furan oxidation

A 0.05 M solution of furan (34 mg, 0.5 mmol) in methanol (10 mL) was introduced into the electrochemical device (channel dimensions: 3 mm × 30 mm × 100 µm, volume 9 µL) through a syringe pump (flow rate 80 µL/min; residence time: 7 s) with an applied current of 2 mA (current density: 1.11 mA/cm^2^) and collected at the outlet to give **5** after removal of the solvent. The product was identified by GC/MS *m*/*z* (EI): M^+^ 129.1.

#### Kolbe electrolysis

A 0.1 M solution of 2-phenylacetic acid (**6a**) (136 mg, 1 mmol) in acetonitrile (10 mL), with 10 mol % triethylamine (13 µL) was introduced into the electrochemical device (channel dimensions: 3 mm × 30 mm × 127 µm, volume 11 µL) through a syringe pump (flow rate 40 µL/min; residence time: 17 s) with an applied current of 4 mA (current density: 2.22 mA/cm^2^) and collected at the outlet to give **7a** with 40% yield (72 mg, 0.4 mmol). The product was identified by GC/MS *m*/*z* (EI): M^+^ 183.3.

#### General procedure for the synthesis of iodonium salts 11

A solution of aryl iodide **8** (0.1 M) and aryl compound **9** (0.3 M) was prepared in 2 M H_2_SO_4_/acetonitrile with 25% acetic anhydride and introduced into the electrochemical device (channel dimensions: 3 mm × 30 mm × 254 µm, volume 23 µl) through syringe pumps (flow rate 80 µL/min; residence time: 17 s) with an applied current of 30 mA (current density: 16.67 mA/cm^2^) and collected at the outlet. The solvent was then removed, water was added (3 mL) and the iodide was precipitated by addition of KI (166 mg, 1 mmol) to give the diaryliodonium salts **11**.

#### Di-*p*-tolyliodonium iodide (11a) [24]

Collection of 3 mL, yield: 100 mg (72%); colourless solid; mp 162–164 °C; ^1^H NMR (400 MHz, CDCl_3_) δ 2.27 (s, 6H), 7.08 (d, *J* = 8 Hz, 4H), 7.74 (d, *J* = 8.3 Hz, 4H); HRMS–EI (*m*/*z*): [M − I]^+^ calcd for C_14_H_14_I, 309.0135; found, 309.0130.

#### (4-Ethylphenyl)(*p*-tolyl)iodonium iodide (11b) [25]

Collection of 5 mL, yield: 114 mg (51%); colourless solid; mp 156–158 °C; ^1^H NMR (400 MHz, CDCl_3_) δ 1.13 (t, *J* = 7.6 Hz, 3H), 2.27 (s, 3H), 2.56 (q, *J* = 7.6 Hz, 2H), 7.09 (m, 4H), 7.77 (m, 4H); HRMS–EI (*m*/*z*): [M – I]^+^ calcd for C_15_H_16_I, 323.0291; found, 323.0297.

#### (4-Isopropylphenyl)(*p*-tolyl)iodonium iodide (11c) [24]

Collection of 5 mL, yield: 139 mg (60%); colourless solid; mp 154–157 °C; ^1^H NMR (400 MHz, CDCl_3_) δ 1.14 (d, *J* = 6.9 Hz, 6H), 2.28 (s, 3H), 2.81 (td, *J* = 7.0, 13.9 Hz, 1H), 7.11 (m, 4H), 7.76 (m, 4H); HRMS–EI (*m*/*z*): [M – I]^+^ calcd for C_16_H_18_I, 337.0448; found, 337.0440.

#### Phenyl(*p*-tolyl)iodonium iodide (11d) [24]

Collection of 5 mL, yield: 93 mg (44%); colourless solid; mp 135–139 °C; ^1^H NMR (400 MHz, CDCl_3_) δ 2.27 (s, 3H), 7.12 (d, *J =* 8.1 Hz, 2H), 7.30 (m, 2H), 7.48 (t, *J* = 7.4 Hz, 1H), 7.77 (dd, *J* = 2.0, 8.7 Hz, 2H), 7.88 (dd, *J* = 1.0, 8.3 Hz, 2H); HRMS–EI (*m*/*z*): [M – I]^+^ calcd for C_13_H_12_I, 294.9978; found, 294.9973.

#### (4-Ethylphenyl)(phenyl)iodonium iodide (11e)

Collection of 5 mL, yield: 82 mg (39%); colourless solid; mp 159–161 °C; ^1^H NMR (400 MHz, CDCl_3_) δ 1.14 (t, *J* = 7.6 Hz, 3H), 2.57 (q, *J* = 7.7 Hz, 2H), 7.12 (d, *J* = 8.4 Hz, 2H), 7.29 (t, *J* = 7.7 Hz, 2H), 7.44 (t, *J* = 7.4 Hz, 1H), 7.79 (d, *J* = 8.3 Hz, 2H), 7.88 (d, *J* = 7.5 Hz, 2H); ^13^C NMR (100 MHz, CDCl_3_) δ 14.9, 28.6, 117.5, 121.1, 131.1, 131.2 (2C), 131.5 (2C), 134.5 (2C), 134.8 (2C), 148.1; HRMS–EI (*m*/*z*): [M – I]^+^ calcd for C_14_H_14_I, 309.0140; found, 309.0130.

#### (4-Isopropylphenyl)(phenyl)iodonium iodide (11f)

Collection of 5 mL, yield: 88 mg (19%); colourless solid; mp 146–148 °C; ^1^H NMR (400 MHz, CDCl_3_) δ 1.15 (d, *J* = 6.9 Hz, 1H), 2.82 (td, *J* = 6.9, 13.8 Hz, 1H), 7.14 (d, *J* = 8.4 Hz, 2H), 7.30 (t, *J* = 7.8 Hz, 1H), 7.44 (t, *J* = 4.4 Hz, 1H), 7.79 (d, *J* = 8.4 Hz, 2H), 7.89 (d, *J* = 7.6 Hz, 2H); ^13^C NMR (126 MHz, CDCl_3_) δ 23.6 (2C), 34.0, 117.4, 121.2, 129.9 (2C), 131.1, 131.5 (2C), 134.6 (2C), 134.7 (2C), 152.7 ppm; HRMS–EI (*m*/*z*): [M – I]^+^ calcd for C_15_H_16_I, 323.0291; found, 323.0298.

#### (4-(*tert*-Butyl)phenyl)(phenyl)iodonium iodide (11g)

Collection of 5 mL, yield: 148 mg (64%); colourless solid; mp 162–164 °C; ^1^H NMR (400 MHz, CDCl_3_) δ 1.23 (s, 9H), 7.34 (m, 4H), 7.48 (t, *J* = 7.4 Hz, 1H), 7.83 (m, 2H), 7.92 (td, *J* = 1.7, 2.9 Hz, 2H); ^13^C NMR (100 MHz, CDCl_3_) δ 30.0 (3C), 34.1, 116.5, 120.4, 127.8 (2C), 130.0, 130.4 (2C), 133.4 (2C), 133.7 (2C), 153.7; HRMS–EI (*m*/*z*): [M – I]^+^ calcd for C_16_H_18_I, 337.0448; found, 337.0451.

#### *m*-Tolyl(*p*-tolyl)iodonium iodide (11h) [24]

Collection of 5 mL, yield: 78 mg (36%); colourless solid; mp 85–89 °C; ^1^H NMR (400 MHz, CDCl_3_) δ 2.27 (s, 6H), 7.09 (d, *J* = 8.1 Hz, 2H), 7.19 (m, 2H), 7.65 (d, *J* = 7.9 Hz, 1H), 7.72 (s, 1H), 7.79 (d, *J* = 8.8 Hz, 2H); HRMS–EI (*m*/*z*): [M – I]^+^ calcd for C_14_H_14_I, 309.0135; found, 309.0141.

#### (4-Ethylphenyl)(*m*-tolyl)iodonium iodide (11i)

Collection of 5 mL, yield: 57 mg (25%); colourless solid; mp 124–127 °C; ^1^H NMR (400 MHz, CDCl_3_) δ 1.14 (t, *J* = 7.6 Hz, 3H), 2.28 (s, 3H), 2.57 (q, *J* = 7.6 Hz, 2H), 7.12 (d, *J* = 8.5 Hz, 2H), 7.21 (m, 2H), 7.67 (d, *J* = 7.9 Hz, 1H), 7.74 (s, 1H), 7.80 (d, *J* = 8.4 Hz, 2H); ^13^C NMR (100 MHz, CDCl_3_) δ 13.9, 20.4, 27.6, 116.1, 119.7, 130.2, 130.3 (2C), 130.6, 131.2, 133.6 (2C), 133.9, 141.1, 147.1; HRMS–EI (*m*/*z*): [M – I]^+^ calcd for C_15_H_16_I, 323.0291; found, 323.0298.

#### (4-Isopropylphenyl)(*m*-tolyl)iodonium iodide (11j)

Collection of 5 mL, yield: 43 mg (18%); colourless solid; mp 152–155 °C; ^1^H NMR (400 MHz, CDCl_3_) δ 1.14 (d, *J* = 6.9 Hz, 2H), 2.27 (s, 3H), 2.81 (td, *J* = 6.9, 13.8 Hz, 1H), 7.13 (d, *J* = 8.4 Hz, 2H), 7.20 (m, 2H), 7.68 (d, *J* = 8.0 Hz, 1H), 7.75 (s, 1H), 7.80 (d, *J* = 8.4 Hz, 2H); ^13^C NMR (100 MHz, CDCl_3_) δ 20.4, 22.6 (2C), 32.9, 116.0, 119.6, 128.8 (2C), 130.1, 130.7, 131.0, 133.6 (2C), 134.0, 140.9, 151.4; HRMS–EI (*m*/*z*): [M – I]^+^ calcd for C_16_H_18_I, 337.0448; found, 337.0444.
